# Supplementary material for: Nanopore sequencing data analysis using Microsoft Azure cloud computing service
Source: PLoS One. 2022 Dec 2;17(12):e0278609. doi: 10.1371/journal.pone.0278609 (PMC9718390; doi:10.1371/journal.pone.0278609)
Supplement: S1 File — (PDF) [file pone.0278609.s001.pdf]

Nanopore sequencing data analysis using Microsoft Azure cloud computing service

Linh Truong[1]

[1]PathWest Laboratory Medicine WA

1 Works for me

Linh Truong

ABSTRACT

This protocol provides instruction to set up the analytic pipeline to process raw data from Oxford Nanopore Sequencing. This pipeline leverages the computing resources available in Microsoft Azure cloud server as well as hospital site at Fiona Stanley Hospital. The raw data in FAST5 format would be converted to FASTQ format, demultiplexed, renamed to appropriate sample ID and filtered based on pre-determined quality threshold. The QC plots would also be generated for ongoing monitoring purposes of sequencing output and quality. The entire data flow from the hospital premise to the cloud and vice versa is completely automated and secured.

PROTOCOL INFO

Linh Truong . Nanopore sequencing data analysis using Microsoft Azure cloud computing service. **protocols.io**
https://protocols.io/view/nanopore-sequencing-data-analysis-using-microsoft-chm6t49e

FUNDERS ACKNOWLEDGEMENT

Microsoft Australia
Grant ID: Microsoft Partner of the year

CREATED

Oct 10, 2022

LAST MODIFIED

Oct 19, 2022

PROTOCOL INTEGER ID

71070

Section 1: Generation of data on-site

1 Load the multiplexed HLA library pool consisting of 48 individuals onto a MinION flow cell. The data is acquired using MinKNOW software for 16 hours using default settings. 16h

MinION
Sequencer
Oxford Nanopore Technologies MinION 1B / MinION 1C

16:00:00

2 The raw FAST5 files are stored in a local folder on the MinION-connected PC.

MinION-connected PC
Computer
Dell N/A
Intel® Core™ i&-7700K CPU @ 4.20Ghz, 32 GB RAM, 64-bit operating system and GPU driver NVIDIA GTX 1080 Ti

3 An automation agent for Loome Integrate runs on the MinION-connected PC and checks for new FAST5 files every 30 minutes.

Loome Integrate
by BizData Pty Ltd

Section 2: Data migration to Microsoft Azure

4 The input files are automatically uploaded by the Loome Integrate agent into a container in an Azure blob storage account, deployed within the PathWest Azure subscription. The files are uploaded using Transport Layer Security (TLS), and are encrypted at rest using 256-bit AES encryption.

AzCopy upload

```
azcopy copy <local_folder> <remote_container> --recursive
```

Command to upload data to Azure using the AzCopy command-line tool (https://learn.microsoft.com/en-us/azure/storage/common/storage-use-azcopy-v10).

Section 3: Orchestration of analysis pipeline in Microsoft Azure

5 The Loome Integrate agent detects that the sequencing job has been completed when it finds a file named "final_summary_<GUID>.txt", and then triggers a new job to deploy the necessary resources and to start the processing steps using the Azure Batch service.

Loome Integrate
by BizData Pty Ltd

6 Loome communicates with the Azure Batch service and tells it to run the analysis using a Docker container that is automatically pulled by Azure Batch from a private Azure Container Registry in PathWest's Azure subscription.

Azure Batch service
by Microsoft

Section 4: Workflow in the cloud server

7 1h 16m 25s

Azure Batch automatically deploys a GPU-enabled Virtual Machine (VM) for basecalling, de-multiplexing, quality trimming and QC overview using the following commands.

Guppy basecaller

```
guppy_basecaller --input_path XX --save_path XX --flowcell FLO-MIN111 --kit SQK-109 --device cuda:0
```

01:07:10 (representative runtime)

Guppy barcoder

```
guppy_barcoder --input_path XX --save_path XX --config configuration.cfg --device cuda:0 --records_per_fastq 0 --trim_barcodes
```

00:03:06 **(representative runtime)**

Concatenate & rename file

```
cd /each_barcode_folder
cat *.fastq > barcodeXX.fastq
```

00:00:30 **(representative runtime)**

NanoFilt

```
cat barcodeXX.fastq | NanoFilt -q 7 -l 500 >
barcodeXX_sampleID.fastq
```

00:05:39 **(representative runtime)**

8 When each of the VMs was running, the input data is copied into their local disk for faster processing, run the analyses, and then copied the results back into blob storage so that the VMs could be deleted when processing had been completed. Loome Integrate, in coordination with Azure Batch, orchestrates these steps.

AzCopy download

```
azcopy copy <remote_container> <local_folder> --recursive
```

Command to download data from Azure using the AzCopy command-line tool (https://learn.microsoft.com/en-us/azure/storage/common/storage-use-azcopy-v10).

AzCopy upload

```
azcopy copy <local_folder> <remote_container> --recursive
```

Command to upload data to Azure using the AzCopy command-line tool (https://learn.microsoft.com/en-us/azure/storage/common/storage-use-azcopy-v10).

9 Loome Integrate detects the completion of all tasks in the Azure Batch job and sends an email

to notify that the analysis has been successfully completed or to report an error.

Loome Integrate
by BizData Pty Ltd

Section 5: Data migration from Microsoft Azure server

10 If the analysis has been successfully completed, the Loome Integrate agent downloads the results in FASTQ format into the MinION-connected PC.

Loome Integrate
by BizData Pty Ltd

AzCopy download

```
azcopy copy <remote_container> <local_folder> --recursive
```

Command to download data from Azure using the AzCopy command-line tool (https://learn.microsoft.com/en-us/azure/storage/common/storage-use-azcopy-v10).

Section 6: Final analysis of results

11 The demultiplexed FASTQ file is analysed by a commercial HLA allele assignment software, GenDX NGSengine.

NGSengine
by GenDX

The HLA alleles are curated by laboratory staff for accuracy and suitability to reporting.
